# Cellular human tissue-engineered skin substitutes investigated for deep and difficult to heal injuries

**DOI:** 10.1038/s41536-021-00144-0

**Published:** 2021-06-17

**Authors:** Álvaro Sierra-Sánchez, Kevin H. Kim, Gonzalo Blasco-Morente, Salvador Arias-Santiago

**Affiliations:** 1 Cell Production and Tissue Engineering Unit, Virgen de las Nieves University Hospital, Andalusian Network of Design and Translation of Advanced Therapies, Granada, Spain; 2 Biosanitary Institute of Granada (ibs.GRANADA), Granada, Spain; 3 Department of Dermatology, University of Arkansas for Medical Sciences, Little Rock, AR USA; 4 Department of Dermatology, Virgen de las Nieves University Hospital, Granada University, Granada, Spain; 5 Department of Dermatology, Faculty of Medicine, University of Granada, Granada, Spain

**Keywords:** Translational research, Tissue engineering

## Abstract

Wound healing is an important function of skin; however, after significant skin injury (burns) or in certain dermatological pathologies (chronic wounds), this important process can be deregulated or lost, resulting in severe complications. To avoid these, studies have focused on developing tissue-engineered skin substitutes (TESSs), which attempt to replace and regenerate the damaged skin. Autologous cultured epithelial substitutes (CESs) constituted of keratinocytes, allogeneic cultured dermal substitutes (CDSs) composed of biomaterials and fibroblasts and autologous composite skin substitutes (CSSs) comprised of biomaterials, keratinocytes and fibroblasts, have been the most studied clinical TESSs, reporting positive results for different pathological conditions. However, researchers’ purpose is to develop TESSs that resemble in a better way the human skin and its wound healing process. For this reason, they have also evaluated at preclinical level the incorporation of other human cell types such as melanocytes, Merkel and Langerhans cells, skin stem cells (SSCs), induced pluripotent stem cells (iPSCs) or mesenchymal stem cells (MSCs). Among these, MSCs have been also reported in clinical studies with hopeful results. Future perspectives in the field of human-TESSs are focused on improving in vivo animal models, incorporating immune cells, designing specific niches inside the biomaterials to increase stem cell potential and developing three-dimensional bioprinting strategies, with the final purpose of increasing patient’s health care. In this review we summarize the use of different human cell populations for preclinical and clinical TESSs under research, remarking their strengths and limitations and discuss the future perspectives, which could be useful for wound healing purposes.

## Skin wound healing

Skin is a vital organ with multitude of functions, one of which is to serve as a barrier to protect against external agents that can cause serious harm. Its relevance becomes apparent with extensive loss of skin due to deep injuries or burns, which affect many parts of human body (limbs, back, and trunk). Delayed intervention can lead to chronic wounds generation or fluid and electrolyte imbalance, poor thermal regulation and sepsis that can ultimately cause death^1^.

**Fig. 1:**
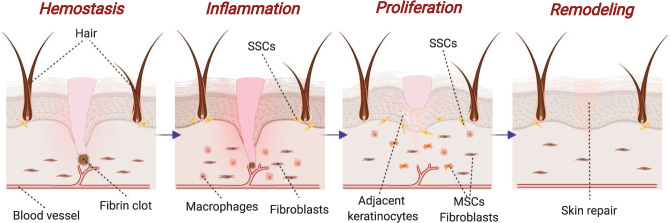
Phases of skin wound healing process. Hemostasis: activation of fibrin is responsible of clot formation and bleeding is stopped. Inflammation: damaged cells are phagocyted and factors are released to provoke cell migration and proliferation. Proliferation: cells such as dermal fibroblasts, MSCs and SSCs (mesenchymal and skin stem cells) achieve wound’s site and form a provisional extracellular matrix. Remodeling: collagen fibers are realigned, and residues are removed. Created with BioRender.com.

To avoid these undesired outcomes, a complex but well-orchestrated process divided in four overlapping phases (hemostasis, inflammation, proliferation, and remodeling) called wound healing (Fig. 1), plays a crucial role after a cutaneous injury, restoring function and appearance of damaged skin with minimal scarring^2^.

This process requires the involvement and coordination of many cell types and signaling pathways^3^. Firstly, vasoconstriction is achieved due to endothelin and factors released by injured cells, such as epinephrine and catecholamines, and moreover, platelets produce platelet-derived growth factor (PDGF), which activates mesenchymal cells from smooth muscles in the vessel walls causing contraction^3,4^. To conclude hemostasis, platelets, through G protein-coupled receptor, bind to thrombogenic subendothelial matrix^5^, activating integrins (αIIbβ3 or α2β1) and glycoproteins (Ib-IX-V and VI), which increase the attachment to fibrinogen, fibronectin and von Willebrand factor and between platelets (platelets plugs)^6,7^. Finally, platelets within the plug releases many growth factors (PDGF, transforming growth factor β-TGF-β-, or epidermal growth factor -EGF-), required for the next stages and moreover, provide a surface for assembly and activation of coagulation complexes lead by Factor X, and where, after Factor XIII crosslinks fibrin, thrombus is formed serving as provisional wound matrix^3,8^.

In inflammatory phase, transcription-independent pathways (Ca^2+^ waves, reactive oxygen species gradients, and pyrogenic molecules) and damaged associated factors such as H_2_O_2_ are responsible of inflammatory cells recruitment, activating keratinocytes regeneration and promoting new vessel formation^3,9^. In particular, neutrophils secrete antimicrobial agents and phagocyte bacteria and cell debris, meanwhile, macrophages have a microbicidal and pro-inflammatory effect at the beginning, but then, develop an anti-inflammatory role, which accelerate wound healing through the formation of new vessel (Tie2+) and the release of vascular endothelial growth factor (VEGF)^3,10^. Moreover, they participate in proliferation phase; inducing the transition of dermal fibroblasts into myofibroblasts and depositing collagen and other extracellular matrix (ECM) components, and also in re-epithelization and remodeling; releasing proteases and phagocytizing excessive cells and matrix no required^3,8,10^. Mast cells are also important for wound contraction because synthesized enzymes chymase and tryptase, as well as histamines and VEGF, which stimulates keratinocyte proliferation and re-epithelialization and enhances fibroblast proliferation and collagen synthesis^3,11^.

Proliferation phase is also triggered by many different cell types^12^. The most important are endothelial cells, which are responsible of angiogenesis in response to factors such as VEGF, PDGF, TGF-β, and fibroblast growth factor (FGF). This is regulated by Notch pathways through VEGF-A produced by subcutaneous adipose stromal cells^3,13^. In addition, fibroblasts synthesize ECM and express genes that are responsible of its proliferation and migration, and myofibroblasts, transient cells derived from local fibroblasts and others cells such as mesenchymal stem cells and epithelial cells, also deposit ECM and exhibit contractile characteristics; processes that are fundamental for wound healing^3^.

Finally, regeneration of the dermis is favorable due to their fibrous nature, allowing for migration and proliferation of macrophages and fibroblasts necessary for remodeling and promoting connective tissue formation^3,10^. In the case of epidermal layer, re-epithelialization is a complicated process where keratinocytes located in the wound edge loss their adhesions and express integrins, which leads to increased Erk-MAPK signaling and inflammatory cytokine synthesis, causing hyperproliferation of keratinocytes and immune cell activation^3,14^. In addition, human skin stem cells (hSSCs) migrate from their niches in order to replace the lost keratinocytes^8,12,15^ and also express higher levels of integrins (α2β1, α3β1, α6β4), that binds collagen or laiminin^3^, and release growth factors, which participate in generation of epithelial cells like keratinocytes, promoting re-epithelization of injured skin^12,16–18^.

On balance, wound healing is a coordinated and complex process where many factors such as, inflammatory skin diseases, deep injuries, large sized or chronic wounds can provoke a deregulation due to an altered immune response and the lack of local adult skin cells and hSSCs available for migration, which causes problems to achieve a correct homeostatic restoration^15,19,20^.

When these critical cells are lacking due to deep and difficult to heal wounds, human mesenchymal stem cells (hMSCs) can also contribute to re-epithelization^21^ by stimulating collagen production and reducing fibrosis and scar formation by releasing many growth factors such as EGF or basic fibroblast growth factor (bFGF)^12^.

Hence, much of the efforts have been dedicated to understanding the mechanisms of wound healing and to develop clinically viable therapies based on tissue engineering, to help patients restore function of damaged skin.

## Tissue engineering and material science of skin

Tissue engineering (TE) is an interesting and growing multidisciplinary field that involves several biomedical areas such as cell biology, material science, engineering or medicine. It appears as a necessity to solve the lack of organ donors or another efficient substitute for the organ required. For this reason, TE tries to manufacture artificial organs and tissues under controlled conditions to be transplanted in vivo in those cases where own patient’s regenerative or reparative capacities are not achieved^22^.

Study of TE strategies requires to evaluate many aspects such as cell sources, cell nature, material science, incorporation or not of growth factors and disease models required (injuries and animals). Regarding cell biology, selection of an appropriate cell type will depend on the target tissue but the main challenge for clinical use will be to select among allogeneic (stem cells included) or autologous source due to the advantages and disadvantages associated to each one^22–24^. The other important aspect in TE is material science; first approaches were based on the use of synthetic biomaterials that provided structural support and replaced organs but without functionality^23^. However, research of ECM has provoked the development of new biomaterials capable of resembling biological and mechanical aspects such as three-dimensional (3D) structures (scaffolds), which enable nutrient’s transport and vascularization^22–24^. Considering their nature, biomaterials could be synthetic, naturally derived or acellular tissue matrices and they must be biocompatible, biodegradable and bioresorbable to be replaced by native tissue without rejection^23^.

In the case of skin, a tissue-engineered skin substitute (TESS) is any safe product, constituted of human cells and bio-scaffolds, capable of replacing damaged human skin and resembling its structural and functional characteristics such as flexibility, protective barrier or transepidermal water loss^25,26^.

In most TESSs, the cellular component is composed of human adult keratinocytes and fibroblasts as part of epidermal and dermal layers, respectively^20^. However, due to the many advantageous properties^8,27^ of human stem cells (hSCs) and the specific role of hSSCs and hMSCs in restoring homeostatic conditions^12,15^, new approaches in the field of skin engineering are focusing on the incorporation of these cell types to TESSs^21^ (Table 1).

**Table 1:** Different cell types used for tissue-engineered skin substitutes (TESSs) considering clinical studies.

	Human cell type used for TESSs	Ease of isolation	Possibility to differentiate into different cell types	Time required to treat patients	Possibility of immune rejection	Proven Safety	Ethical issues	Proven effectiveness
Human adult skin cells	Keratinocytes	-One or two skin biopsies (3–9 cm^2^) -Specific conditions for culture (feeder layers or commercial media)	No	Autologous use: 7–95 days	No	Yes	No	Yes, although with limitations
				Allogeneic use: 0–24 days	Yes	No	Yes	More clinical studies are required
	Fibroblasts	-One or two skin biopsies (3–9 cm^2^)	No	Autologous use: 7–95 days	No	Yes	No	Yes, although with limitations
				Allogeneic use: 0–24 days	Yes	Yes	Yes	Before the development of composite skin substitutes were extensively used
	Melanocytes	-One or two skin biopsies (3–9 cm^2^) -Specific conditions for culture (commercial media) -Difficult to isolate	No	Autologous use: 30–95 days	No	No (risk of cancer)	No	More clinical studies are required
	Langerhans cells and Merkel cells	-Skin biopsies -Difficult to isolate	No	–	–	–	No	Non-clinical studies using these cells for wound healing
Human stem cells	Skin stem cells	-Skin biopsies -Difficult to isolate	Yes (in vitro and in vivo)	–	–	–	Yes Proliferative capacity of stem cells	Non-clinical studies using these cells for wound healing
	Induced pluripotent stem cells	-Any human adult cell	Yes (in vitro and in vivo)	–	–	–	Yes Proliferative capacity of stem cells Genetic manipulation	Non-clinical studies using these cells for wound healing
	Mesenchymal stem cells	-Bone marrow: iliac crest injection -Wharton’s Jelly: umbilical cord sample -Adipose tissue: adipose tissue biopsy or liposuction	Yes (in vitro and in vivo)	0–28 days	No for autologous source Yes, for allogeneic source. Although due to their immunomodulatory properties risk is reduce	Yes	Yes Proliferative capacity of stem cells Although they have been used (autologous or allogeneic source) for other diseases	More clinical studies are required

Several biomaterials such as collagen^28^, chitosan^29^, elastin^30^, or hyaluronic acid^31,32^ have been used for manufacturing TESSs. On balance, they differ in their internal structure: porous, fibrous, hydrogel, or ECM nature, which provide advantageous and drawbacks depending on therapeutic purposes^33^.

In this review, we analyze the different cell types, human adult skin cells but also human stem cells, used to develop research models of TESSs, at preclinical or clinical environment, for the treatment of deep and difficult to heal wounds.

## Human adult skin cells in TESSs

Human skin is composed of several cell types distributed in the different layers of skin: epidermis, dermis and hypodermis. Epidermis is mainly composed of keratinocytes, but also melanocytes, Langerhans cells and Merkel cells are present. Dermis is primarily constituted by fibroblasts and extracellular matrix, meanwhile, hypodermis is mainly comprised of adipose tissue cells.

**Fig. 2:**
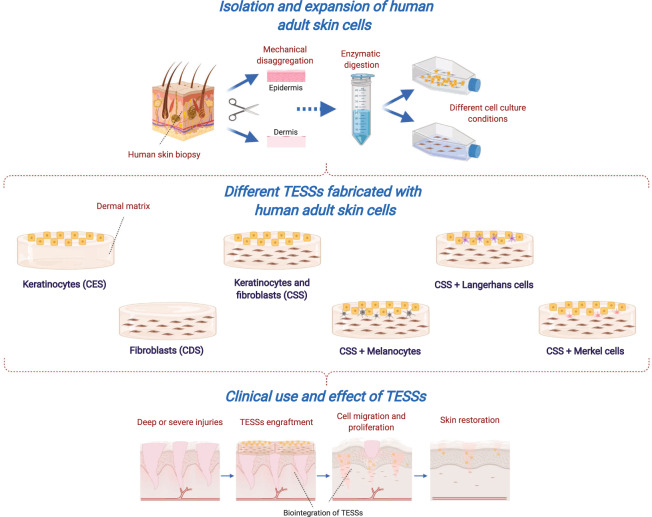
Tissue-engineered skin substitutes (TESSs) fabricated with human adult skin cells and their role in wound healing process. After a deep, severe or chronic injury where, normal phases of healing are not possible, fabrication of TESSs from cells of a human skin biopsy is the most usual advanced therapy. Keratinocytes, fibroblasts and the rest of epithelial cells are isolated, expanded and used in combination with a biological matrix to produce sheets of cultured epithelial substitutes (CESs), cultured dermal substitutes (CDSs) and composite skin substitutes (CSSs), which are engrafted to promote and facilitate cell activation and the release of growth factors necessary to achieve reparation, regeneration and homeostasis of skin. Created with BioRender.com.

Most of the non-commercial substitutes studied are constituted by epidermal, dermal or both layers, where keratinocytes and fibroblasts are the most used cell types; however, some researchers explored the use of the other epithelial cell types with the purpose of fabricating TESSs that better resemble native skin (Fig. 2).

### Human keratinocytes for preclinical TESSs

Keratinocytes were the first skin cell type isolated and explored^34^, and for this reason the former models of TESSs were based on these cells only. Development of cultured epithelial substitutes (CESs) for burn patients has been one of the main objectives, which has led to many extensively studied commercial devices^35,36^.

In recent years, the number of studies evaluating the use of human keratinocytes-only TESSs have been limited. Some authors have used these CESs to compare different culture techniques^37^ or dermal matrices (ECM derived from fibroblasts)^38^. The necessity of including dermal components to support in vivo proliferation and preservation of keratinocytes was early demonstrated in athymic mice by Rennekampff et al.^39^ where human keratinocytes transplanted with an acellular dermal matrix onto full-thickness skin defects, developed a fully differentiated epidermis and persisted in all animals grafted (vs. 63.6% of those animals without a dermal component).

In 2019, Horch et al.^40^ studied in vivo the use of keratinocyte monolayers in hyaluronic acid membranes demonstrating that when keratinocytes were directly implanted towards the full-thickness wound bed of athymic mice, formation of a multilayered and differentiating epidermis was faster (14 days) than conventional technique (>21 days).

### Human keratinocytes for clinical TESSs

Owing to the important role of epidermis as a protective barrier, CESs with keratinocytes were the first TESSs explored in patients (Table 2). Since 1981, many clinical studies have analyzed the role of CESs in skin regeneration^41–56^, mainly for the treatment of burns (14 of 16 studies), although treatment of surgical wounds was also reported.

**Table 2:** Clinical use of human cultured epithelial substitutes (CESs).

References	Cells	Type of clinical study	*N* (male / female)	Age (years)^a^	Treatment-related adverse events	Indication	Total body surface area (TBSA) affected (%)^a^	Affected area covered (%)^a^ or Affected area covered (%TBSA)^a^	TESS successful engraftment (%)^a^ or TESS successful engraftment (% TBSA)^a^	Period between skin biopsy and grafting (days)^a^	Follow-up (months)^a^	Outcomes
^41^	Autologous epidermal cells	Case Report	2 (2/0)	49.5 ± 16.2 (38–61)	None	Burns	60 ± 28.3 (40–80)	–	100	14–21	6	Absence of stratum corneum during the first week, but 6 months after grafting, the epidermis could not be distinguished from native skin
^42^	Autologous keratinocytes	Case Report	17 (11/6)	31.8 ± 14.4 (2–56)	None	Burns	56.1 ± 18.3 (31–85)	–	31	18–22	1–1.5	In some cases, 80% of epithelialized skin was achieved after 6 weeks. Hypertrophic scar formation was less than observed in comparable areas treated with meshed grafts
^43^	Autologous keratinocytes	Case Report	26	-	None	Burns	50 (2–75)	–	15 (0–98)	7	–	Limited success
^44^	Autologous keratinocytes	Case Report	26	29.03 ± 18.7 (5–80)	None	Burns	33 ± 16.7 (10–75)	12.5 ± 6.4 (5–35)	32.1 ± 15.3 (0–65)	20–22	6	Keratinocyte culture “take” was significantly lower than that split-thickness skin grafts
			4	3.7 ± 1.9 (1–5)	None	Giant congenital nevus	–	55.9 ± 30.9 (30–100)	56.2 ± 13.7 (40–70)			
^45^	Autologous keratinocytes	Case Report	16	29.7 (10–56)	None	Burns	68.2 (42–85)	15.9 (4–59)	4.7 (0–18.6)	—	4.3 (1.7–9.2)	No impact on the definitive closure of massive burn wounds
^46^	Autologous keratinocytes	Case Report	5 (4/1)	38.8 (20–60)	None	Burns	59.6 (48–70)	—	93.6 (87–100)	—	48	Only two patients required any additional grafts to cover open wounds that had originally been covered with CESs
^47^	Autologus epithelial cells	Case Report	28	35.3	None	Burns	52.2	10.4 (2–35)	26.9	—	60	After 5 years of use, CES engraftment was unpredictable and inconsistent. Should be used only as biologic dressing and experimental adjunct to conventional burn wound coverage with split-thickness autograft
^48^	Autologous keratinocytes	Observational Study	29	26	None	Burns	77	—	53	14–18	3	Good cosmetic appearance when compared with meshed split-thickness skin grafting
			8	2.8	None	Scald wounds	23	—	73			
^49^	Autologous keratinocytes	Case Report	7 (4/3)	11.6 ± 10.7 (1.7–30)	None	Burns	45.7 ± 19.88 (20–75)	—	97.1 ± 7.5	14	2–20	Epidermal regeneration evaluated 1 month after grafting was stable and complete. Epidermis appeared fully differentiated with a well-developed stratum granulosum
^50^	Autologous keratinocytes	Non-randomized trial	8	10.5 ± 2	Reconstructive procedures were required in the first 2 years for functional problems	Burns	92.5 ± 1.9	44 ± 7	60 ± 8	21	24	After follow-up scars had a significantly smoother surface and less pigmentation than traditional meshed autografts
^51^	Autologous keratinocytes	Non-randomized controlled trial	7	32.1 ± 18 (9–65)	None	Burns	68.7 ± 20.4 (51–95)	—	65 ± 46.6	16–22	1	An undulated dermo-epidermal junction was present underneath the grafted epithelia cultured on fibrin gel matrices
^52^	Autologous epidermal cell sheets	Non-randomized controlled trial	14	40.8 ± 18.3 (3–61)	None	Palmoplantar wounds	—	100	100	1	27.6 ± 10.5 (12–48)	Expression of keratin 9 was continuously observed after the transplantation
^53^	Autologous keratinocytes	Non-randomized controlled trial	7	6.4 ± 1.4	None	Burns Reconstructive releases	75.9 ± 5.0	—	100	—	12	Successful vascularization was observed in 45.7 ± 14.2% of the wounds, after 14 days
^54^	Autologous epidermal cells	Case Report	2 (2/0)	33 ± 1.4 (32–34)	None	Burns	75 ± 14.1 (65–85)	—	0	21–28	0.4	These substitutes could be useful as temporary biological dressing as the take was so poor
^55^	Allogeneic keratinocytes	Retrospective Observational Study	13 (7/6)	62.2 ± 15.7 (34–84)	None	Chronic skin ulcers	—	100	100	—	3.2 ± 2.3 (0.6–7)	There was an overall reduction of 91.5% of wound size in comparison with initial value
^56^ (NCT00832156)	Autologous epidermal cells	Randomized Controlled Trial (parallel assignment)	40 (25/15)	50 ± 19 (20–85)	None	Burns	24.2 ± 13 (6–51)	—	90 ± 12.6 (6–51)	13	12	Epithelialization increased in the wounds after 5–7 days (71.2 ± 24.8%)

^a^Expression of measures: mean ± standard deviation (range).

Regarding to culture and manufacturing process of CESs, in most of former studies reviewed (in chronological order), keratinocytes were expanded and isolated by enzymatic detachment from culture flasks and directly engrafted onto patients^41–48,50,52,54^. In these cases, results differed depending on: parameters analyzed, endpoint of follow-up or pretreatment strategy, but, in general, studies that only applied this type of CES reported worse results in terms of graft take, due probably to the effect of digestive enzymes on epidermal cells.

For this reason, some authors concluded that their use could be interested as temporary biological dressing^54^ or combined with meshed split-thickness skin grafting^42,43,48^. In other cases, the previous engraftment of artificial^52^ or allogeneic split-thickness skin grafts^43,46^ improved the take of these conventional CESs and accelerate wound healing^52^ due to the increase of capillarity density^43^.

However, due to the risk associated with these strategies, researches started to investigate the fabrication of more complex CESs where epithelial cells and biomaterials were cultured in vitro before engraftment^49,51,53,55,56^. Two studies evaluated the use of fibrin and in both cases take of grafts was higher, improving the relation cost-efficiency^49^ and demonstrating that fibrin facilitated the formation of dermo-epidermal junction because ECM proteins secreted by autologous keratinocytes were retained^51^.

Sheridan et al.^53^ and Pajardi et al.^55^ developed CESs based on acellular dermis or membranes, respectively. In the first case^53^, vascularization after 14 days was higher in the case of autografts (98 ± 1% vs. 45.7 ± 14.2%), however, results of Vancouver Scar Scores (VSSs) after 12 months demonstrated that no differences existed between autologous CESs (1.2 ± 0.7) and autografts (1.0 ± 0.4). In the second case^55^, at the end of the follow‐up, a reduction of 91.5% of wound dimensions was achieved with allogeneic CESs.

Other biomaterials used were collagen and elastin^56^ but, in this case, autologous CESs were engrafted before gold standard treatment (autografts) was applied. Results revealed that the combination of CESs and autografts increased epithelization against those cases where only autografts were applied (71% vs. 67%), and after 12 months of follow-up, Patient and Observer Scar Assessment Scale (POSAS) reported better results when CESs were grafted (14.2 ± 7.2 vs. 18.4 ± 10.2). This was the only randomized controlled clinical trial reviewed (NCT00832156)^56^.

Interestingly, one of the studies demonstrated the importance of using an appropriate skin autograft or source. Yamaguchi et al.^52^ compared three different treatments for palmoplantar wounds: (i) CESs with autologous epidermal cells from palmoplantar sites, (ii) no-palmoplantar skin grafts, and (iii) palmoplantar skin grafts. No expression of keratin 9 was observed in the case of no-palmoplantar skin grafts and after 1 year, wound size was higher (26.77 ± 6.72 cm^2^) than in those patients treated with CESs (12.27 ± 4.14 cm^2^) and palmoplantar skin grafts (4.24 ± 0.68 cm^2^).

On balance, studies using CESs have evolved from the first reported, including new culture techniques and strategies, however, it does not seem to be the best alternative when deep wounds or difficult to heal wounds needs to be treated. To date, a total of 259 patients (29.1 ± 17.2 years old), the majority of them with burn injuries (88.0% of the cases), with a mean of 57.4 ± 20.0% total body surface area (TBSA) affected, have been treated using this strategy, and the percentage of successful engraftment was 60.5 ± 35.0% without adverse events except in one case, where more reconstructive procedures were required for functional problems^50^ (Table 2).

### Human fibroblasts for preclinical TESSs

Development of human cultured dermal substitutes (CDSs) is essential to achieve a proper integration of the engraftment and successful wound healing. Mineo et al.^31^ developed a dermal substitute composed of hyaluronic acid, collagen and human dermal fibroblasts. They demonstrated in vitro, increased amount of VEGF and hepatocyte growth factor (HGF), which effectively created a vascularized wound bed for autologous skin grafting in Sprague Dawley rats with deep dermal burns.

Other studies explored the role of extracellular matrix of human fibroblasts to support the growth of these cells and develop more natural substitutes^57,58^ for full-thickness wounds, demonstrating in vitro and in vivo, on Sprague Dawley rats, that they have notable effects on wound healing, facilitating fibroblast infiltration, collagen bundle production, and elastic fiber and blood vessel formation^58^.

Mohd Hilmi et al.^29^ evaluated a chitosan sponge matrix seeded with human dermal fibroblasts, engrafted onto full-thicknesses wounds excised on the irradiated skin of Sprague Dawley rats. Wounds treated with chitosan CDS showed the most re-epithelialization level (33.2 ± 2.8%) and scar size of wounds were significantly decreased compared with control group where duoderm CGF was applied (0.13 ± 0.02 cm vs. 0.45 ± 0.11 cm).

Finally, the addition of other cell types such as endothelial cells could be useful to increase the regeneration potential of CDSs. One study fabricated a TESS based on endogenous matrix produced by human dermal fibroblasts and cultured with human fibroblasts and endothelial cells, which were capable of forming capillary-like-structures effectively anastomosed with host vessels in vivo^59^.

### Human fibroblasts for clinical TESSs

With the advancement of culture techniques and ability to isolate dermal fibroblasts, clinical studies have evaluated the use of CDSs for the treatment of chronic skin ulcers (7 of 10 studies), surgical wounds and burns (Table 3)^55,60–68^. Important findings from these studies highlight the release of cytokines or growth factors, which activates many pathways for skin regeneration^60,63^.

**Table 3:** Clinical use of human cultured dermal substitutes (CDSs).

Reference	Cells	Type of clinical study	*n* (male / female)	Age (years)^a^	Treatment-related adverse events	Indication	Total body surface area (TBSA) affected (%)^a^	Affected area covered (%)^a^ or affected area covered (%TBSA)^a^	TESS successful engraftment (%)^a^ or TESS successful engraftment (% TBSA)^a^	Period between skin biopsy and grafting (days)^a^	Follow-up (months)^a^	Outcomes
^60^	Allogeneic fibroblasts	Case Report	5 (4/1)	59.5 ± 19.5 (39–81)	In one patient, infection after 14 days was observed but resolved	Burns	–	100	87.8 ± 9.6 (75–98)	0 (TESSs were cryopreserved previously)	10–14	Failed to take permanently on the wound surface, but was able to produce cell growth factors which improved wound healing
			1 (0/1)	88	None	Necrotizing fasciitis	–	100	88			
^61^	Allogeneic fibroblasts	Case Report	3 (2/1)	58.6 ± 12.3	None	Skin ulcers prior to autologous skin grafting	–	100	26.8	0 (TESSs were cryopreserved previously)	6	A greater amount of healthy granulation tissue was produced and suitable for autologous skin grafting
^62^	Allogeneic fibroblasts	Case Report	13 (3/10)	65 ± 9.5 (48–79)	One case presented local infection	Chronic and consecutive leg ulcers	–	100	81.3 ± 9.65 (61–96)	0 (TESSs were cryopreserved previously)	2 ± 1.2 (0.75–4.75)	Effective not only for producing tissue granulation and epithelialization, but also for removing necrotic tissue
^63^	Allogeneic cryopreserved or fresh fibroblasts	Case Report	7 (5/2)	40.4 ± 16.6	None	Surgical wounds	–	100	100	0 (for TESSs previously cryopreserved) 7 (considering only time of culture of the fresh TESSs)	0.27	Cryopreserved TESSs were capable of releasing sufficient amounts of several cytokines and of promoting re-epithelialization to a degree comparable to fresh TESSs
^64^	Allogeneic fibroblasts	Case Report	5 (0/5)	62.6 ± 24.1 (37–89)	None	Skin ulcers	–	100	66.7	0 (TESSs were cryopreserved previously)	2	Capable of promoting wound healing in intractable skin ulcers that failed to improve despite daily treatment with bFGF for more than 2 months
^65^	Allogeneic fibroblasts	Case Report	8 (3/5)	53.6 ± 14.1 (33–70)	One case presented local infection	Intractable skin ulcers	–	100	78.4 ± 20.5 (36–100)	0 (TESSs were cryopreserved previously)	1 ± 0.3	Healthy granulation tissue and epithelization developed rapidly in many cases
^66^	Autologous fibroblasts	Randomized, Controlled, Multicenter Clinical Trial	31 (21/10)	61.2 ± 11.4	None	Diabetic ulcers	–	100	84	21–28	12	Time required for complete healing were lower in the TESS group than control group
^67^	Autologous fibroblasts	Prospective, Open‐Labeled Clinical Trial	5 (5/0)	60.6 ± 11.1 (47–73)	30 adverse events, two directly related to the treatment but resolved	Diabetic ulcers	–	100	94 ± 8.9	>10	3	Side effects were not serious, and three patients were completely healed within 12 weeks after application
^55^	Allogeneic fibroblasts	Retrospective Observational Study	17 (11/6)	63.3 ± 14.2 (42–91)	None	Chronic skin ulcers	–	100	73.0	–	3.2 ± 2.3 (0.6–7)	There was an overall reduction of 73% in comparison with the initial wound size
^68^	Allogeneic fetal fibroblasts	Randomized, Double-Blind, Phase I Clinical Trial	10 (9/1)	29.5 ± 11 (13–46)	None	Surgical wounds	–	100	94	7 (considering only time of culture of the TESSs)	0.5	Re-epithelialization was faster than in control groups

^a^Expression of measures: mean ± standard deviation (range).

In most of the cases, CDSs were fabricated using allogeneic fibroblasts^55,60–65,68^. Cells were cultured over different scaffolds and placed cell‐seeded side down onto the wound surface.

Regarding to the type of clinical studies reviewed, three were considered as clinical trials^66–68^, one was an observational study^55^ and remaining were classified as case reports^60–65^.

Among these, three studies compared different therapies^63,66,68^. Yamada et al.^63^ evaluated the use of allogeneic fresh or cryopreserved fibroblasts cultured on a bilayer sponge composed of hyaluronic acid and collagen for the treatment of deep surgical wounds, demonstrating that cryopreserved cells were capable of releasing cytokines and promoting re-epithelialization at the same level as fresh cells. You et al.^66^ reported better results in terms of complete ulcer healing when compared the use of hyaluronic acid-based autologous CDSs (84%) and non-adherent foam dressings (34%). Finally, Momeni et al.^68^ studied the use of amniotic membranes alone or combined with allogeneic fibroblasts and compared the results with a control therapy (Vaseline gauze). Results revealed that wound closure of surgical wounds was higher when amniotic membranes were used (alone— 95.5%—, with fibroblasts—94%—vs. control—59%—) and re-epithelialization was faster (alone—11.3 ± 2.9 days—, with fibroblasts—10.1 ± 2.4 days—vs. control —14.8 ± 1.6 days—).

Interestingly, a combination of hyaluronic acid and collagen was the preferred matrix for the manufacture of CDSs^60–65^. Different wounds were treated using these allogeneic CDSs (burns^60^, surgical wounds^63^, and ulcers^61,62,64,65^), but similar results indicated that their application would be interested as biological wound dressing to produce granulation tissue and secrete VEGF, bFGF, and ECM proteins useful for mesh-auto skin grafts.

Finally, Morimoto et al.^67^ used an artificial dermis and autologous fibroblasts for the treatment of diabetic ulcers demonstrating an important wound size reduction after 21 days (from 7.1 ± 4.9 cm^2^ to 3.4 ± 2.3 cm^2^). In contrast, Pajardi et al.^55^ used hyaluronan as dermal matrix for the treatment of chronic ulcers reporting a wound size reduction of 73%.

One-hundred five patients (63 males and 42 females), older than those treated with CESs (58.4 ± 14.7 years old), were treated with CDSs. Small injuries (87.6% of cases were chronic skin ulcers) were evaluated and successful engraftment was achieved in 79.5 ± 20.0% of the cases. Adverse events were only observed in five cases, related to local infections (Table 3).

### Combination of human keratinocytes and fibroblasts for preclinical TESSs

In recent years, the combination of human keratinocytes and fibroblasts in TESSs, called composite skin substitutes (CSSs) has been explored.

CSSs resemble normal skin by containing an epidermal layer of autologous or allogeneic keratinocytes and a dermal layer of fibroblasts incorporated into a stromal scaffold. They not only provide structural dermo-epidermal support, but also deliver growth factors (EGF, PDGF, VEGF) and extracellular matrix that increase the rates of recovery and healing^69,70^.

For these reasons, many studies have looked different strategies to explore potential benefits of CSSs. A composite TESS manufactured with fibrin-hyaluronic acid biomaterial has been recently evaluated in vivo in immunodeficient mice with excisional wounds and compared with another fibrin-agarose CSS and secondary wound healing dressings, demonstrating favorable outcomes, similar to autografts in terms of clinical (POSAS scale results: eight for CSS vs. six for autografts), homeostasis (transepidermal water loss: 6.42 ± 0.75 g/h/m^2^ for CSS vs. 6.91 ± 1.28 g/h/m^2^ for autografts) and histological restoration, after eight weeks of engraftment^32^.

Similarly, Tissue Biology Research Unit of Zurich developed human CSSs based on type I collagen hydrogels, demonstrating, in vitro and in vivo in full-thickness skin defects of athymic rats, that these TESSs homogeneously developed a well-stratified epidermis over the entire surface of the grafts and displayed a well-defined basal cell layer where keratin 19/keratin 15-double-positive keratinocytes are essential in growing skin^71,72^.

Supp et al.^73^ manufactured composite TESSs based on collagen-glycosaminoglycan for studying recessive dystrophic epidermolysis bullosa (RDEB) on immunodeficient mice and demonstrated that formation of structurally normal anchoring fibrils appears to require expression of type VII collagen in both skin layers. Bacakova et al.^74^ also developed collagen-based CSSs by utilizing a nanofibrous poly-l-lactide and observed cell migration and proliferation after 14 days of in vitro culture.

Interestingly, Centre de recherche en organogénèse expérimentale de l’Université Laval/LOEX developed a self-assembly approach, which allows for the production of a scaffold-free cell-based CSS^75,76^. Briefly, the dermal layer is composed of stacked fibroblast sheets and keratinocytes are seeded onto the tissue, forming a stratified and cornified epidermis. Auger and Germain’s group have optimized this protocol and studied this model in vitro and in vivo (athymic mice with full-thickness skin injuries), demonstrating timely production of CSSs that could improve clinical availability for the effective wound coverage of patients^77–80^.

In addition, these types of TESSs have been used as a research tool to investigate other pathological conditions and learn more about the role of these treatments in wound healing. For instance, bin Busra et al.^81^ demonstrated in vivo in mice, that fibrin-based CSSs enhanced healing of irradiated wounds after radiotherapy, with higher expression of TGF-β1, PDGF and VEGF than monolayer substitutes.

The vascularization of CSSs has also been studied by incorporating human endothelial cells^82–85^, with reports showing improvement of graft survival and the formation of vascular networks, which physically resemble normal wound healing process.

### Combination of human keratinocytes and fibroblasts for clinical TESSs

Clinical benefits of CESs and CDSs have been observed in many patients; however, the most studied TESSs have been CSSs composed of human keratinocytes and fibroblasts that have been used for the treatment of several dermatological pathologies since 1989 (Table 4)^86–106^.

**Table 4:** Clinical use of human composite skin substitutes (CSSs).

References	Cells	Type of clinical study	*n* (male/ female)	Age (years)^a^	Treatment-related adverse events	Indication	Total body surface area (TBSA) affected (%)^a^	Affected area covered (%)^a^ or Affected area covered (%TBSA)^a^	TESS successful engraftment (%)^a^ or TESS successful engraftment (% TBSA)^a^	Period between skin biopsy and grafting (days)^a^	Follow-up (months)^a^	Outcomes
^86^	Autologous keratinocytes and fibroblasts	Case Report	4 (3/1)	33.5 ± 15.5 (20–53)	None	Burns	51.5 ± 15.4 (40–74)	–	69.2	21.2 ± 3.5 (19–28)	1	There was an improved quality of skin healed with cultured cells
^87^	Autologous keratinocytes and fibroblasts	Case Report	2 (2/0)	40.5 ± 14.8 (30–51)	None	Burns (1)	81	100	–	21	10	Mature epidermis and well-differentiated papillary and reticular dermis were formed
						Excised wounds (1)	–	100	100			
^88^	Autologous keratinocytes and fibroblasts	Prospective Randomized Clinical Study	17	12.7 ± 3.3 (1–50)	Increased incidence of exudates	Burns	68.8 ± 2.4 (51–87)	–	0 (5 patients) 50–90 (12 patients)	25.3 ± 9.3	12	Pigmentation was greater, scar was less raised, but regrafting was more frequent in skin substitutes compared with split-thickness autografts
^89^	Autologous keratinocytes and fibroblasts	Case Report	5 (4/1)	15.6 (4–38)	None	Burns	77.8 (58–87)	–	100	>14	6	Connection between epidermis and connective tissue, together with spontaneous repigmentation was observed
^90^	Allogeneic keratinocytes and fibroblasts	Prospective Randomized Compared Clinical Study	11	–	None	Surgical wounds	–	–	90	–	2	Rapid healing and reduction of the pain
^91^	Autologous keratinocytes and fibroblasts	Case Report	3 (3/0)	4.33 (2–7)	None	Burns	72 (63–88)	12.8 (8.4–21.3)	100	21	2	Stable epithelium covered a layer of newly formed fibrovascular tissue. Smooth, pliable, and hypopigmented skin
^92^	Autologous keratinocytes and fibroblasts	Prospective, Randomized, Non-blinded Clinical Study	45 (34/11)	10.6 ± 1.6	None	Burns	64.6 ± 2	16.7 ± 2.6	95.4 ± 1.8	28	12 ± 1	Healed skin was soft, smooth, and strong with irregular pigmentation. Impact of TESS on wound closure increases proportionately with the magnitude of the wound area
^93^	Allogeneic keratinocytes and fibroblasts	Case Report	3 (2/1)	36.3 ± 14.9	Local inflammation	Burns	<20	–	23.5 ± 10.7	17–24	0.25	By 1 week after grafting there remained a few islands of keratinocytes on an inflamed bed
^94^	Autologous keratinocytes and fibroblasts	Case Report	2 (2/0)	22.5 (17–28)	None	Burns	67.5 (50–85)	–	100	24.6 (23–26)	24	Epidermal regeneration was stable, with good cosmetic outcome
^95^	Autologous keratinocytes and fibroblasts	Randomized Controlled Trial	40	-	None	Burns	73.4	–	81.5 ± 2.1	–	12	Vancouver Scale Scores were not different for erythema, pliability, or scar height, but pigmentation remained deficient against autograft treatment
^96^	Autologous keratinocytes and fibroblasts	Case Series	20	22.9 ± 16.3 (6–62)	None	Burns (13) Giant nevus (5) Graft-vs.-host disease (1) Neurofibromatosis (1)	–	–	Burns: 56.9 ± 25.3 (10–90) Giant nervus: 82 ± 7.6 (70–90) Graft-vs.-host disease: 90 Neurofibromatosis: 75	25	1–18	Epithelization obtained was permanent
^97^ (NCT00718978)	Autologous keratinocytes, melanocytes and fibroblasts	Single Group Assignment Open-Label Clinical Trial	11 (3/8)	24.6 (2–57)	None	Giant nevus (5) Tumors (2) Scars (3) Trauma (1)	–	–	30–95	30	36	Loss of the epithelial layer varied markedly (from 5 to 70%) while fibroblast cellular component growth prevailed
^98^	Autologous keratinocytes and fibroblasts	Multicenter Retrospective Observational Cohort Study	25 (23/2)	29 ± 11 (9–58)	Thirteen patients presented wound infection (*P. aeruginosa* mainly)	Burns	74 ± 17 (35–100)	24 ± 13 (7–60)	49 ± 30 (0–100)	23 ± 5 (12–28)	45 ± 27 (2–91)	Characteristic scarring of mesh interstices was avoided. Epithelialization was observed
^99^	Autologous keratinocytes and fibroblasts	Prospective Uncontrolled Case Study	5 (1/4)	55.2 ± 18.5 (26–74)	None	Skin ulcers	–	100	100	52–63	6–19	Effective treatment of long-standing hard-to-heal venous or mixed ulcers
^100^	Autologous keratinocytes and fibroblasts	Case Report	4 (1/3)	42.3 ± 14.7 (29–63)	None	Burns	64.8 ± 26.9 (40–98)	–	94.8 ± 4.3 (90–100)	21	1–9	Dermal part had a well-vascularized dermal matrix and bilayer structure was conserved
^101^	Autologous keratinocytes and fibroblasts	Case Report	1 (1/0)	48	None	Burns	40	–	88	19	1	CSS completely covered the wound area and smoothly adapted to the wound ground. Color resemblance of the transplant to the healthy skin increased through the follow-up period
^102^	Autologous keratinocytes and fibroblasts	Case Report	2	9.5 ± 6.3 (4–14)	None	Burns	80 ± 21.2 (65–95)	–	–	–	36	Appearance of the skin did not differ significantly from the areas treated with autografts
^103^	Autologous keratinocytes and fibroblasts	Case Report	1 (0/1)	29	None	Burns	70	100	100	28	6	Patient was discharged after 163 days of hospital admission with a complete skin coverage, correct functioning of the four limbs and autonomous walking
^104^ (NCT00591513)	Autologous keratinocytes and fibroblasts	Prospective Randomized Open-Label Paired-Site Comparison Clinical Trial	16 (14/2)	6.3 ± 1.1 (1.4–17.5)	None	Burns	79.1 ± 2.2 (59.5–95.9)	33.4 ± 3.5 (9.7–71.6)	83.5 ± 2.0	32.1 ± 1.1 (24–42)	12	Vascularization of the dermal component occurred during the first week after grafting, and CSS stabilized barrier function, basement membrane, and nutrient supply were restored
^105^	Autologous keratinocytes and fibroblasts	Case Series	14 (12/2)	34 ± 16 (10–63)	None	Burns	74 ± 13 (52–92)	19±15 (3–53)	98 ± 5 (85–100)	62.7 ± 4.8 (56–71)	38 ± 23	No loss of the epithelium was observed during the first-year post-intervention or reported subsequently. Grafted TESSs expanded when the patient grew or gained weight.
^106^	Autologous keratinocytes and fibroblasts	Phase I Two-armed, Open-Label Prospective Clinical Trial	10 (6/4)	9 ± 4 (7–14)	Four cases of hematoma	Burns (1) Reconstructive surgery for burn scars (9)	–	100	67 ± 32 (0–100)	32 ± 4 (26–38)	15 ± 7 (2–25)	Three months postoperatively, there was a multilayered, well-stratified epidermis and a dermal compartment comparable to native skin

^a^Expression of measures: mean ± standard deviation (range).

Most of the studies evaluated the use of CSSs for the treatment of burns, however, experimental designs differed from cell populations used (autologous [19]^86–89,91,92,94–106^ vs. allogeneic [2]^90,93^), biomaterials selected, randomization or not, comparison or not with other treatments or pretreatment required.

In those cases where different treatments were compared, engraftment of autografts was the gold standard treatment used as reference for each patient^88,90,92,93,95,101,102,104–106^. In one of the cases where allogeneic cells were used, CSSs did not take and the engraftment of more autografts was required^93^. Rest of comparative studies, reported positive results for CSSs in terms of percentage of TBSA closed ([20.5 ± 2.5% for CSSs vs. 52.1 ± 2% for autografts^95^], [29.9 ± 3.3% for CSSs vs. 47.0 ± 2% for autografts^104^] after 28 days in both cases), time of healing (7.4 ± 0.9 days for CSSs vs. 7.9 ± 1.5 days for autografts^90^), appearance (scars were less raised than autografts^88^), percentage of wound area closed (95.4% for CSSs vs. 99% for autografts after 28 days^92^), manipulation (easy in comparison with CESs^102^), protein expression (keratin 19 and type IV collagen^105^) or percentage of epithelialization (63.5 ± 35% after 21 days of engraftment^106^).

Remaining studies indicated the beneficial role of using CSSs alone^89,96,98–100,103^ or combined with autografts^86,87,91,94,97,101^ for the treatment of deep and difficult to heal injuries, evaluating different parameters such as graft take, histological appearance of new skin and cosmetic and functional outcomes.

Interestingly, some researches remarked the importance, as in the case of CESs, of a pretreatment with auto-dermis or allo-dermis to increase clinical benefits of CSSs^86,91,94,98,106^.

Regarding to the biomaterials used for the fabrication of the scaffolds, different types of collagen^87,90,93,95,101,106^ or combined with glycosaminoglycan^86,88,89,91,92,104^ were the preferred sources, although different formulations of plasma/fibrin were also reported^94,96,98,102,103^. Finally, hyaluronic acid^97^ or acellular dermal matrices derived from human fibroblasts^99,100,105^ were evaluated too.

To date, a total of 241 patients (25.6 ± 14.9 years old) with severe burns (81.7% of the cases), surgical wounds or skin ulcers with a mean of 69.2 ± 11.1% of TBSA affected have benefited from CSSs with a mean percentage of successful engraftment of 80.2 ± 26.3% (0%-100%) with slight adverse events such as local inflammation or increased incidence of exudates (Table 4). Interestingly, eleven of twenty one studies included children and five studying children exclusively^91,92,102,104,106^.

### Human melanocytes for preclinical TESSs

In order to develop a TESS that most resemble natural skin, the incoporation of other cell types present in epidermal layer, such as melanocytes, has been evaluated in preclinical stages.

Liu et al.^107^ were one of the first groups to develop a TESSs composed of human fibroblasts, melanocytes and keratinocyes in a type I collagen gel. In vitro and in vivo results revealed proper integration, morphology and successful repair of skin defects in athymic mice and black skins were observed by 6 weeks after grafting.

Biedermann et al.^108,109^ also developed a pigmented skin composite based on collagen, which was transplanted onto full-thickness skin wounds in rats. After 3 weeks, blood vessels, but no nerve fibers or lymphatic vessels were observed^108^. However, peripheral host nerve fibers were found 15 weeks after transplantation^109^. The same group studied the inflammatory response of these pigmented substitutes, which revealed that granulocytes infiltrate the entire graft at 1 week post-transplantation, while monocyte/macrophage recruitment was observed at 3–12 weeks^110^.

Boyce’s laboratory also evaluated the use of human melanocytes in collagen-based TESSs. They analyzed in vitro and in vivo (athymic mice) the incorporation of different densities of cryopreserved and recovered human melanocytes in a human CSS. Melanocytes were localized into the dermal–epidermal junction of skin substitutes and were capable of restoring cutaneous pigmentation and ultraviolet photoprotection after full‐thickness skin loss conditions^111^, which was corroborated by Goyer et al.^112^, regardless of whether light or dark pigmentation phototype melanocytes were used^113^.

### Human Langerhans and Merkel cells for preclinical TESSs

Langerhans cells are a specialized population of dendritic cells that are found in the stratum spinosum of epidermis of the skin. They help to drive protective immune responses following infection of the skin^114^. Merkel cells constitute a unique population of postmitotic cells scattered along the dermo-epidermal junction. These cells have synaptic contacts with somatosensory afferents and play a crucial role in sensory discernment^115^. The number of studies evaluating the use of these cell types for TESSs is limited.

Isolation of Langerhans cells (LCs) is a complicated process. For this reason, only two studies have reported the use of in vitro-derived LCs from monocytes^116^ or an acute myeloid leukemia cell line, MUTZ-3^117^, for fabrication of collagen-based TESSs. In both cases, substitutes were composed of human fibroblasts, keratinocytes, and derived LCs. After 11–14 days of in vitro culture, histological evaluation featured a fully stratified epidermis with all the characteristic epidermal strata. Langerin-positive cells were detected suprabasally within the epidermis indicating that keratinocytes provide environmental conditions for long-time maintenance of derived LCs.

Other related studies have reported the presence of Langerhans cells (CD1a+ and human leukocyte antigen—HLA+) in vitro or in vivo in TESSs when epithelial cells were incorporated^118–121^. This indicates that isolation of keratinocytes, are likely to contain a small proportion of cells that are LCs.

In the case of Merkel cells, only Hahn et al.^122^ have reported on immunodeficient mice, the presence of host nerve cells and Merkel cells (keratin 20+ − K20+ – and HLA+) from grafted human keratinocytes and fibroblasts in a collagen-glycosaminoglycan scaffold, suggesting that fine touch sensation may be restored after TESS’s engraftment.

## Human stem cells (hSSCs) in TESSs

**Fig. 3:**
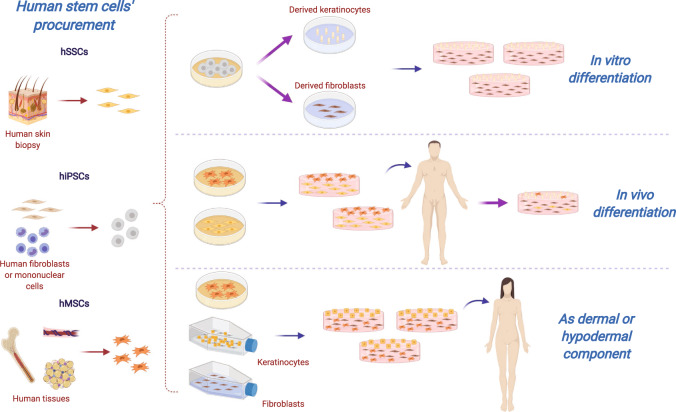
Human stem cells’ (hSCs) strategies for tissue-engineered skin substitutes (TESSs). Different sources of hSCs could be (i) differentiated in vitro to the main cutaneous lineages and then, uses to fabricate artificial skin; (ii) embedded directly into dermal scaffolds and engrafted to achieve an in vivo differentiation; or (iii) combined with human keratinocytes and fibroblasts to benefit from their own angiogenic and immunomodulatory properties. hSSCs human skin stem cell, hiPSCs human-induced pluripotent stem cells, hMSCs human mesenchymal stem cells. Created with BioRender.com.

Human stem cells such as hSSCs, induced pluripotent stem cells (hiPSCs) and hMSCs have been investigated for therapeutic use to enhance wound healing^21,27,123^. This has led to the fabrication of more complex models of TESSs (Fig. 3), which could stimulate more rapid and complete healing; furthermore, drive expression of additional phenotypes to correct anatomic deficiencies through activation of biological signaling pathways^27^.

### Human skin stem cells (hSSCs) for preclinical TESSs

The skin is an attractive source of stem cells because of their abundant supply, easy accessibility, ease of harvesting, and possibly providing immune-privileged cells^124^. hSSCs, characterized by their quiescence state (CD71^-^, EGF-R^low^) and their strong adhesion capacity (high expression of integrin markers)^125^, have been isolated from different parts of skin such as dermal papilla or hair follicles, among others^12,124,126,127^. In particular, dermal papilla stem cells (DPSCs), also called dermal hMSCs, have similar characteristics and differentiation capacity as hMSCs from other tissues^127^, and for this reason they are the hSSCs most studied for skin regeneration and wound repair.

Jeremias et al.^128^ integrated skin-derived hMSCs with different dermal substitutes (Integra^®^ and Pelnac^TM^) and showed that both were able to support the maintenance and growth of skin-derived hMSCs. Salerno et al.^129^ also evaluated the use of these cells in dermo-epidermal skin substitutes constituted of dermal membranes of chitosan, polycaprolactone and a polymeric blend and, after 14 days of in vitro culture, were capable of observing fibronectin deposits, as a result of dermal differentiation.

Relationship between human DPSCs and hair follicle stem cells (FSCs) has been studied in TESSs by constructing a composite based on a porcine acellular matrix with DPSCs and FSCs in the dermal and epidermal layers, respectively. This composite, when grafted in nude mice with full-thickness skin wounds, resulted in successful integration (less contraction), vascularization (higher expression of VEGF), and the presence of DPSCs-induced formation of hair buds (hair‐specific keratin 6—K6hf+)^130^.

Another study developed by Higgins et al.^131^ compared human dermal fibroblasts, DPSCs and FSCs within a collagen scaffold. In vitro and in vivo experiments on nude mice revealed that both, DPSCs and FSCs, can replace interfollicular fibroblasts in skin constructs. Regarding basement membrane formation, DPSCs were found to be superior to fibroblasts with an increased type IV collagen and VEGF expression, coinciding with a formation of a more robust and uniform basal lamina.

Apart from FSCs being used in combination with DPSCs, where a correct stratification, differentiation and well-ordered epithelia was observed^130^, Mohd Hilmi et al.^132^ reported that a chitosan-TESS composed of fibroblasts and FSCs serving as the epidermal component, was capable of restoring rat skin after radiation exposure by an increasing collagen bundle deposition.

The role of dermal hMSCs and other types of hSSCs in wound healing has been compared with human adipose tissue-derived MSCs (hAT-MSCs)^133,134^. Michalak‑Micka et al.^133^ fabricated different collagen-based TESSs constituted of human keratinocytes as epidermal layer and stromal cells from different sources. These substitutes were evaluated in vivo in an immune-incompetent rat model and results revealed that all types of transplants exhibited a multilayered stratified epidermis with a thick stratum corneum. However, an enhanced expression of tropoelastin (a soluble precursor of elastic fibers) were only observed in skin grafts containing hAT-MSCs and dermal hMSCs, which correlated with in vivo results of Zomer et al.^134^, demonstrating their potential to accelerate wound healing.

### Human-induced pluripotent stem cells (hiPSCs) for preclinical TESSs

Human-induced pluripotent stem cells (hiPSCs) are stem cells generated from individual somatic cells by exogenous expression of several transcription factors to initiate the reprogramming process^135^. In skin regeneration and for TESSs, hiPSCs have been successfully obtained from fibroblasts^136–138^ or cord blood mononuclear cells (CBMCs)^139^, and differentiated into fibroblasts and keratinocytes^136–139^.

Itoh et al.^136^ described one of the first models of skin substitute using hiPSCs for the treatment of RDEB. They differentiated embryoid bodies generated from hiPSCs into fibroblasts and keratinocytes and showed that the hiPSCs-derived fibroblasts were capable of producing and secreting mature type VII collagen in addition to expressing other collagen types (I, III, and IV). Moreover, they fabricated and engrafted in mice a TESS constituted of collagen I matrix and these hiPSCs-derived fibroblasts, demonstrating their capacity to support functional and terminal differentiation of human keratinocytes by the expression of K1 and loricrin. In all, they were able to fabricate a complete hiPSCs-derived skin composite, histologically like normal human skin.

The same group was also able to differentiate hiPSCs into melanocytes^137^ and demonstrate that hiPSCs-derived keratinocytes, which expressed K1 and K14, were capable of internalizing melanosomes, essential to generate a functional epidermal-melanin unit.

To support these results, Petrova et al.^138^ focused on hiPSCs-derived keratinocyte differentiation and developed a physiological purification method, which resulted in higher yield isolation of a cell population similar to normal human keratinocytes expressing K14 and p63. After their characterization, these derived cells were used in an in vitro TESS model demonstrating their capacity to form the same structure as the human epidermis and also, develop endoplasmic reticulum Ca^2+^ store, essential for normal keratinocyte signaling and differentiation.

To improve the survival of skin grafts and avoid immune rejection, CBMCs have emerged as a potential cell source for regenerative medicine and hiPSCs. One advantage of CBMCs as a source is the mandatory HLA typing during the CBMC banking process, making available valuable HLA-matched research materials that can be obtained easily^139^. Kim et al.^139^ successfully differentiated hiPSCs from CBMCs into fibroblasts and keratinocytes. These cells were used to produce 3D skin organoids, and after being implanted onto surgical excisions in mice, they resembled skin structure with a similar expression of CD73 and CD105 as primary fibroblasts; and involucrin and loricrin (epidermal differentiation markers) were upregulated.

In addition to fibroblasts and keratinocytes, hiPSCs have been differentiated into other cell types such as melanocytes^137^, sensory neurons and Schwann cells^140^. They have been generated in vitro and successfully incorporated into TESSs with the purpose of fabricating more complex, functional and complete skin substitutes, although exhaustive in vivo analysis is still required.

### Human mesenchymal stem cells (hMSCs) for preclinical TESSs

Mesenchymal stem cells are non-hematopoietic multipotent adult progenitor cells that are found in various tissues, including bone marrow, adipose tissue, and umbilical cord. They can be easily harvested and expanded from the different tissues of adult donors, avoiding any potential ethical issues associated with using embryonic stem cells or with genetic manipulations when using hiPSCs. Moreover, their hypo-immunogenic property allows its immediate use as prepared allogeneic cells without significant host reaction^141–144^, although recent studies have indicated that the immune compatibility between donor and recipient is also important because hMSCs are immune evasive rather than immune privileged^145–147^. Their anti-inflammatory capacity^148^ can also be useful in dampening the inflammatory milieu of chronic non-healing wounds and aid in the healing process.

Another beneficial feature of hMSCs is their plasticity to differentiate into both mesenchymal and non-mesenchymal lineages such as ectodermal keratinocyte-like cells (KLCs)^149^, endothelial cells, and different skin appendages, which is being investigated for skin tissue engineering and wound healing therapies^149,150^.

Moreover, the addition of hMSCs to current skin substitute models can potentially promote angiogenesis by the recipient’s endogenous cells via paracrine signaling with VEGF^151^.

#### Human bone marrow-derived MSCs (hBM-MSCs)

hBM-MSCs have been the most studied and the major source of hMSCs. In the skin, hBM-MSCs’ regenerative potential and their ability to differentiate into non-mesenchymal lineages including endothelial cells, keratinocyte-like cells, and skin appendages^152^, have been demonstrated to be useful for wound healing.

He et al.^153^ studied the use of hBM-MSCs and their capacity to differentiate in vitro and in vivo into epidermal and dermal cells. Better differentiation was observed in the case of dermal cells, in vitro. They fabricated TESSs composed of hBM-MSCs in a collagen membrane and implanted them into surgical skin wounds generated on the back of mice. After 21 days, wounds were completely healed and a differentiated epidermis and dermis were observed, demonstrating that hBM-MSCs could differentiate in the inducing microenvironment in vivo.

Ojeh et al.^154^ used hBM-MSCs as dermal component in a CSS model composed of de‐epidermalized dermis with human keratinocytes and compared it with a traditional CSS composed of human fibroblasts and keratinocytes. In vitro results showed that a hBM-MSC model could generate a hyperproliferative epidermis that was well‐differentiated.

hBM-MSCs have also been analyzed as the epidermal layer in fibrin-TESSs with a dermal layer composed of human fibroblasts^152^. This study compared MSCs from different human sources: bone marrow, umbilical cord Wharton’s jelly (hWJ-MSCs) and adipose tissue (hAT-MSCs). In all cases, an epithelial-like layer was formed after the first week of culture, although after four weeks, more stratified epidermis was observed in the case of hBM-MSCs and hWJ-MSCs. Moreover, after in vivo grafting in nude mice with surgical wounds, mesenchymal cell populations, mainly for hAT-MSCs substitutes, induced the generation of up to ten epithelial-like layers after 15 and 30 days, expressing keratin 5, proteoglycans and collagen fibers, but without expression of HLA markers.

#### Human umbilical cord Wharton’s jelly-derived MSCs (hWJ-MSCs)

Perinatal stem cells such as hWJ-MSCs have been shown to have excellent proliferation and differentiation capabilities to be applied in regenerative medicine^155^.

Garzón et al.^156^ studied these cells in vitro and in vivo by using a bioactive 3D heterotypical model comprised of primary cell cultures of hWJ-MSCs and fibroblasts from oral mucosa or skin in a fibrin-agarose-based matrix as stroma substitute. Their results showed that hWJ-MSCs were unable to fully differentiate into epithelial cells in vitro. However, after in vivo grafting onto immunodeficient, athymic mice, they showed expression of epithelial differentiation and functional markers, and stratification into typical epithelial layers.

Ertl et al.^157^ compared hWJ-MSCs with two different human term placenta-derived mesenchymal stem cells (hP-MSCs) in an in vivo full-thickness wound model in mice. All TESSs fabricated with Matriderm^®^+MSCs induced a faster healing and a higher number of blood vessels in the wound when compared to controls (49 ± 6% of wound reduction for TESSs vs. 22 ± 7% of wound reduction for controls). In another study, Shi et al.^158^ employed hWJ-MSCs with skin microparticles in a murine excisional wound repair model to show multi-direction differentiation into newly formed skin and its appendages such as sebaceous glands, hair follicles and sweat glands.

Interestingly, some authors have explored the use of these silk fibroin-based TESSs combined with an injection of hWJ-MSCs at the edge of the wounds in mice. Results of this treatment indicated that collagen dermis organization was more similar to that typically observed in the normal skin of mice and diminished both innate and adaptative immune infiltrates^159^.

#### Human adipose tissue-derived MSCs (hAT-MSCs)

hAT-MSCs are an attractive source for hMSCs-based construction of TESSs for their ease of harvesting and expansion in culture and versatile differentiation potential into non-mesenchymal lineages such as ectodermal KLCs^149,160,161^. Moreover, compared to MSCs from other sources such as the bone marrow, the procurement of hAT-MSCs is associated with lower morbidity and higher yield of cells^155^.

hAT-MSCs for TESSs have been used, in most of the cases, as a dermal component, alone or combined with other cell types. Some in vitro studies evaluated the role of hAT-MSCs as dermal support for human keratinocytes, showing after 7 days of culture, increased collagen IV expression in the epidermal-dermal junction^162^ and enhanced proliferation of human keratinocytes^163^. These results were corroborated with an in vivo study in a third degree burn model generated in rats where the treatment with TESSs composed of hAT-MSCs and human keratinocytes on a human amniotic membrane reported faster wound regeneration and less inflammatory cell infiltration than control groups^164^. These TESSs have been also analyzed in murine models of full-thickness defects, demonstrating their capacity to enhance wound healing rates^165^ promoting angiogenesis and re-epithelization^166,167^.

Furthermore, favorable outcomes have also been observed when hAT-MSCs were combined with other cells to constitute the dermal matrix. TESSs composed of hAT-MSCs co-cultured with human endothelial cells in the dermal layer and human keratinocytes in the epidermal were capable of forming capillary structures in vitro^168^.

Interestingly, pigmented TESSs fabricated with hAT-MSCs and human fibroblasts in the dermal layer were less dark than those manufactured with fibroblasts only, which indicated that cytokines released by hAT-MSCs maintained melanocytes in an immature state where melanin synthesis was decreased^169^.

Another method of delivering hAT-MSCs is using adipose-derived stromal vascular fraction (SVF), which not only contains MSCs, but also endothelial cells and pericytes that are key contributors to vasculature formation. Klar et al.^170,171^ developed a novel pre-vascularized composite skin substitute model by seeding adipose-derived SVF into a 3D fibrin hydrogel, allowing for the formation of vascular networks in the graft prior to transplantation. In this rat full-thickness wound model, there was more efficient engraftment of the transplanted skin substitute due to rapid anastomoses of the graft capillary plexus with the recipient’s vasculature, epidermal regeneration with stratification, and remodeling of the dermis with low graft contraction.

### Human mesenchymal stem cells (hMSCs) for clinical TESSs

Although most of the recent preclinical studies report the use of hAT-MSCs as the main stem cell for TESSs, the number of clinical studies is still limited. Only a small amount of published research has evaluated the use of hMSCs in TESSs as a therapeutic strategy for wound healing (Table 5).

**Table 5:** Clinical use of human mesenchymal stem cells in tissue-engineered skin substitutes (TESSs).

References	Cells	Type of clinical study	*n* (male/ female)	Age (years)^a^	Treatment-related adverse events	Indication	Total body surface area (TBSA) affected (%)^a^	Affected area covered (%)^a^ or Affected area covered (%TBSA)^a^	TESS successful engraftment (%)^a^ or TESS successful engraftment (% TBSA)^a^	Period between skin biopsy and grafting (days)^a^	Follow-up (months)^a^	Outcomes
^172^	Autologous hBM-MSCs	Case Report	1	77	None	Diabetic wounds	–	100	100	0, 7, and 17	1	Wound showed a steady overall decrease in size and an increase in vascularity of the dermis and in the dermal thickness of the wound after 29 days
^173^	Autologous hBM-MSCs	Case Series	20 (9/11)	64.8 ± 20 (22–91)	None	Severe burns (2) Decubitus ulcer (11) Skin ulcers (7)	50 ± 14.1 (40–60) –Burns	–	100	Few weeks	>2	High tissue regenerative ability was observed, the healing mechanism was activated, and therapeutic effects were independent of age or cause
^174^	Autologous hBM-MSCs	Case Report	1 (1/0)	19	None	Burns—Scar excision wounds	>60	–	60	–	24	Contraction of skin was significantly less at the hBMMSC transplantation site than at the control site
^175^	Autologous hAT-MSCs	Case Report	2 (0/2)	48.5 ± 9.2 (42–55)	None	Surgical wounds— Burns	–	100	100	0	7–60	This technique was not recommended routinely, but should be considered for burns patients with contractures affecting cosmetically or functionally challenging areas
^176^(NCT02619877)	Allogeneic hAT-MSCs	Phase II Multicenter Randomized Clinical Trial (parallel assignment-single blind)	22 (14/8) 17 (13/4)–Control Group	59.9 ± 13.3 (26–80) 68.4 ± 9.9 (43–79)	None	Chronic diabetic ulcers	–	100	100	0	3	Complete wound closure was achieved for 82% of patients in the treatment group and 53% in the control group at week 12
^178^	Allogeneic hWJ-MSCs	Randomized Clinical Trial	5	(30–60)	None	Chronic diabetic wounds	–	100	96.7	–	1	After treatment, some patients reported even a decline in pain
^177^	Autologous hAT-MSCs	Prospective Clinical Analysis	6 (3/3)	66.3 ± 9.0	None	Chronic diabetic ulcers	–	100	74.5 ± 32.5	21	3	There was granulation tissue formation starting from 7 days after topical application. After 90 days, a healed and re‐epithelialized tissue was observed

^a^Expression of measures: mean ± standard deviation (range).

Most of the studies reviewed were case reports^172–175^. Some researchers reported the use of hMSCs-based TESSs combined with autograft treatment^174,175^ and in other cases, a comparison between biomaterials with or without hMSCs was evaluated^173,174,176,177^.

Regarding tissue of origin, three studies evaluated the use of autologous hBM-MSCs^172–174^. Vojtassák et al.^172^ showed enhanced wound healing in one patient with chronic diabetic and venous ulcers using a composite graft fabricated with autologous skin fibroblasts on a collagen and hyaluronan membrane in combination with autologous hBM-MSCs injected and placed on the wounds. After 29 days of treatment, an increased vascularization of dermis was observed due to the differentiation potential of hMSCs into endothelial progenitor cells, which produced VEGF and bFGF.

Yoshikawa et al.^173^ studied a MSCs-based treatment of 20 patients with different pathologies whose acellular dermis grafting had previously failed. They combined cultured autologous hBM-MSCs with a collagen sponge, which resulted in a significant improvement of wounds in 18 of 20 patients. Some wounds were treated with collagen membrane only and subcutaneous formation was not observed, in contrast to the rest of wounds treated with hMSCs, where infiltration of inflammatory cells was notable and CD34+ cells (derived from bone marrow) formed vascular endothelia.

Xu et al.^174^ applied a composite graft comprised of autologous hBM-MSCs embedded in decellularized allogeneic dermal matrix overlaid with autologous split-thickness skin graft for the treatment of hypertrophic scars resulting from burn injuries. Results demonstrated a better outcome with reduced contraction as compared to areas treated with split-thickness skin-graft alone.

hAT-MSCs were used in three studies^175–177^: Arkoulis et al.^175^ and Stessuk et al.^177^ combined autologous hAT-MSCs with different dermal matrices (collagen-glycosaminoglycan and plasma, respectively) to treat eight patients with burn injuries or chronic ulcers. In the first case^175^, authors did not recommend the use of this technique routinely because autograft treatment was required, but it could be useful to use it when dealing with highly complex burns patients with contractures affecting cosmetically or functionally challenging areas. Stessuk et al.^177^ reported a total re-epithelialization in 5 of 9 chronic ulcers and a healing rate of 74.6 ± 32.6% after 9 days of treatment. Interestingly, one wound treated with plasma membrane without cells required a re-treatment.

Last study that used hAT-MSCs was a phase II randomized clinical trial (NCT02619877)^176^ where allogeneic hMSCs embedded on a hydrogel were compared with a control group (Mepitel^®^), for the treatment of chronic ulcers. Results revealed that no obvious clinical rejection existed after 12 weeks (higher anti-HLA antibodies expression in 27% of the patients). Regarding to effectiveness, Kaplan–Meier median time to complete wound healing was 28.5 days for treatment group and 63.0 days for control group.

Finally, one study evaluated the use of allogeneic hWJ-MSCs in combination with amniotic membranes for the treatment of chronic ulcers^178^. After 9 days, wound size declined from 70.96 mm^2^ to 3.07 mm^2^ and wound healing rate was of 96.7%. Moreover, patients reported decreased pain after 1 month of clinical follow up.

Overall, a total of 57 patients (54.4 ± 19 years old) with different pathologies such as burns, diabetic wounds or skin ulcers were treated with hMSCs. Burn patients had 50–60% of TBSA affected and overall successful engraftment of TESSs was 90.2 ± 16.3% without adverse events. Interestingly, five of the seven studies investigated used autologous cells (Table 5).

## Future approach: human immune cells in TESSs

As previously described, participation of immune cells, such as neutrophils, macrophages or mast cells, in wound healing of skin is essential due their dual role as pro-inflammatory cells in first stages, and as anti-inflammatory effectors when safety is ensured^3^.

These cells are capable of phagocyte cell debris, synthetize or release several cytokines, which promotes angiogenesis and wound healing (VEGF), activate keratinocyte’s proliferation, and re-epithelialization or induce fibroblasts transition into myofibroblasts to increase ECM and collagen deposition^3,10^. Moreover, recent studies have indicated that immune cells are members of stem cells niches, developing a proactive role in regulating stem cells when tissues are damaged^10,179,180^.

Macrophages and Foxp3+ CD4+ regulatory T (Treg) cells seems to be the most important immune cell populations involved in this context^10,179,180^. Macrophages are capable of sensing the metabolic environment^181^ and therefore, modulating stem cells function^179^, meanwhile, Treg cells infiltrated in wounds express the epidermal growth factor receptor (EGF-R), which is related with an improvement of wound healing^180^.

In wound healing of skin when regenerative phases are triggered, macrophages around the follicle die off and release factors such as WNT7b and WNT10a, which promote the activation of FSCs^182^. Moreover, these macrophages physically contact with epithelial stem cells, secreting pro-proliferative and epithelial remodeling factors such as IL-10 and PDGF-β^183^, apart from TGF-β1, which induces fibroblast proliferation and their differentiation into myofibroblasts^184^.

In the case of Treg cells, they are predominantly localized around hair follicles in contact to FSCs. Their role in skin regeneration could be interested for lesions, which affect epidermal appendages such as hair follicles^179,180^. Several researches have studied the link between Treg cells and hair follicles biology, demonstrating that in alopecia areata patients the number of Foxp3+ Treg cells is reduced in comparison with healthy controls^185^. In addition, stimulation of Treg proliferation with IL-2 administration demonstrated successful hair regeneration in 80% of patients^186^, which is due to the expression of the Notch ligand Jagged‐1 (Jag1), required to promote hair follicle cycling by enhancing the activation and differentiation of FSCs^180^.

For all of this, incorporation of human immune cells in TESSs, alone or combined with other cell populations might be interesting to increase regeneration potential and develop more complex models of artificial skin, which included epidermal appendages such as hair follicles. However, no preclinical research has been published yet in this field, which is essential to ensure their wound healing’s safety and effectiveness.

## Allogeneic cells: a real strategy for clinical TESSs?

In addition to determining the cell composition of TESSs, for clinical purpose, the selection between allogeneic or autologous cells is a critical decision. While the use of allogeneic cells ensures quicker availability, graft survival is usually short-term (4–8 weeks)^187^. The use of autologous source avoids any possible host rejection and permits a more permanent TESS for full-thickness burns and chronic wounds^95^. The downside of autologous cells is the longer period (~4 weeks) required to produce a sufficiently sized graft.

Interestingly, many of the TESSs developed for clinical use (Tables 2–5) were constituted of autologous cells, mainly for CESs, CSSs or hMSC-based TESSs, with only five studies reporting the use of allogeneic cells^55,90,93,176,178^. However, in the case of CDSs, the use of allogeneic fibroblasts was preferred against autologous fibroblasts^66,67^, which could be explained by their use as temporary dressing to prepare the wound´s bed for future therapies or the small size of wounds treated, when a short-term biological recovery dictates the long-term outcomes^147^ (Table 3).

In the case of adult skin cells, the use of autologous populations for TESSs seems to be clear, to avoid rapid rejection. However, when allogeneic hMSCs are selected, there is a controversy because their immunogenicity have been proven^188^, but no acute adverse events have been reported when were applied as therapy in several pathologies^146^.

To avoid this concern, immunosuppression treatment could be effective for the use of allogeneic cells in many clinical conditions, however, this poses a risk for long-term therapies where other pathologies could be developed due to a continuous suppression of immune system and moreover, in the case of skin or TESS transplants, is either less or/not effective^189^.

On balance, although immune rejection of allogeneic hMSCs occurs more slowly than other cell types^145,188^, they are immune evasive rather than immune privileged and for this reason, it could be interesting to use haplo-identical hMSCs^145,147^ to increase the potential benefits of allogeneic TESSs when autologous approach is not possible.

## Main biomaterials for clinical TESSs

Many different biomaterials have been investigated for the development of TESSs; from xenogeneic scaffolds such as porcine acellular matrix^130^, natural polymers like silk fibroin^159^, agarose^32,156^ or chitosan^29,129,132^ to substances that resemble in better way the native dermal components of skin: collagen^56,60–65,86–93,95,101,104,106^, plasma/fibrin^49,51,94,96,98,102,103^, hyaluronic acid^60–65,97^, elastin^56^, amniotic membrane^68,178^ or extracellular matrix derived from fibroblasts^39,99,100,105^.

For clinical purposes, the main biomaterials used have been collagen alone or combined with glycosaminoglycan, hyaluronic acid, plasma/fibrin, amniotic membranes and acellular dermal matrices (Table 6).

**Table 6:** Main biomaterials used for tissue-engineered skin substitutes (TESSs) considering clinical studies.

Biomaterial	Type of clinical TESSs fabricated	Advantages	Drawbacks	References
Collagen	CESs, CDSs, CSSs, and hMSC-based TESSs	Most abundant animal protein High tensile strength and stability	Lack of intrinsic angiogenic properties	^56,60–65,87,90,93,95,101,106,172,173^
Collagen-glycosaminoglycan	CSSs and hMSC-based TESSs	Glycosaminoglycan increases mechanical properties and fibril formation of collagen	Requires cross-linking	^86,88,89,91,92,104,175^
Hyaluronic acid	CDSs, CSSs and hMSC-based TESSs	Ease to handle Biosafety corroborated by its use in cosmetic field Angiogenic properties	Less mechanical properties in comparison with collagen	^55,60–65,97,172^
Plasma/fibrin	CESs, CSSs, and hMSC-based TESSs	Composed of proteins that participate in wound healing Enhances cell proliferation	Combination with other biomaterials is required to increase mechanical properties	^49,51,94,96,98,102,103,177^
Amniotic membrane	CDSs and hMSC-based TESSs	High tensile strength Releases several growth factors for angiogenesis and cell proliferation	Difficult to obtain	^68,178^
Acellular dermal matrix	CESs, CDSs. CSSs, and hMSC-based TESSs	ECM components similar to native human Minimizes the host response	Specific formation is required to obtain and more time	^53,67,99,100,105,174^

*CES* cultured epithelial substitute, *CDS* cultured dermal substitute, *CSS* composite skin substitute, *hMSC* mesenchymal stem cell, *TESS* tissue-engineered skin substitute.

Collagen is the most abundant of animal proteins, localized in soft and hard connective tissues where its fibrils, with high tensile strength and stability via cross-linking, comprise the majority of ECM and form a highly organized, 3D scaffold that surrounds the cells. Moreover, it is a dynamic and flexible biomaterial (used as sponge, gel or membrane) with high biocompatibility and intrinsic biodegradability ideal for biomedical applications^190^. Glycosaminoglycan is a negatively charged polysaccharide and one of the most prevalent crosslinks of collagen, that affects their mechanical properties and fibril formation^191^. The main aspect to consider the incorporation into TESSs is the presence of negatively charged carboxyl and sulfate groups that are responsible of maintaining water in tissues^192^, and therefore, skin barrier.

Hyaluronic acid is also an important component of human skin^193,194^ and its use for TESSs is recommended. It is easy to handle, their biosafety has been corroborated by its use as injectable dermal fillers^195^, and its effectiveness have been demonstrated for skin restoration in terms of hydration and transepidermal water loss^196^, mainly when the water regulation and neoangiogenic boost are relevant issues^197^. Moreover, biodegraded hyaluronic acid, enhances angiogenic pathways and the migration and proliferation of cells^198^.

In the case of plasma/fibrin-based matrices, their use for TESSs have reported positive results due to the presence of many natural components responsible of coagulation and involved in the first stages of wound healing. It has been demonstrated that enhances cell proliferation (keratinocytes mainly), due to the presence of basement membrane proteins such as laminin, collagen or Perlecan^199^.

Finally, amniotic membrane and acellular dermal matrix are the less common biomaterials used for TESSs, however, they also provide structural support and secrete important proteins or factors required for wound healing. Amniotic membrane has high tensile strength due to a structure comprised of an epithelial monolayer, a thick basement membrane and avascular stroma. In addition, it releases several growth factors for angiogenesis, downregulates TGF-β expression, promotes fibroblast differentiation and keratinocytes proliferation, and reduces levels of pain and discomfort experienced by the patients^200^. The use of acellular dermal matrix derived from fibroblast is an interested strategy because the use of natural ECM shares many properties with native human skin and minimizes the host response after transplantation^78^, but specific formation is required to carry out it successfully.

## Discussion

Notwithstanding the tremendous advances in skin tissue engineering, we have yet to construct a complete TESS. Current substitutes are mainly composed of human keratinocytes and fibroblasts, but still lack some of the functional components such as nerves, adnexal structures and pigmentary cells that make up the native skin, and the esthetic and functional outcome is less than ideal.

Moreover, to develop an ideal TESS that could be applied at clinical level, the use of appropriate wound and animal preclinical models is essential. In this review, most of the in vivo studies evaluated excisional or burn wounds in mice or rats. The use of these animals together with other small mammals such as rabbit or guinea pig is due to their cost and easy to handle, however, their anatomical and physiological skin properties and wound healing process differ from the humans (for example, thin epidermis and dermis and heal primarily through wound contraction instead of re-epithelialization)^201^.

After analyzing their properties, many authors suggested that the use of pigs should be the preferred animal model^201–204^. Among similarities reported, epidermis and dermis of human and pig skin are comprised of four and two layers, respectively, without significant differences of thickness, and dermo‐epidermal junction has an undulating appearance^204^. In terms of functionality, permeability is also similar^204^. However, the use of this model is expensive and more difficult due to their size and for this reason the number of studies with human TESSs is limited^205^ or own pig derived cells are used^206,207^.

Considering the types of wounds analyzed, burns or full-thickness skin injuries are the most studied models at preclinical level but also in a clinical environment. Application of commercial biological substitutes have been extensively reviewed^208^ and analyzed^209^ and even the use of cell therapies have reported positive results^210^. However, the development of TESSs constituted of different cell types and biomaterials seems to be essential to increase skin wound healing potential.

Apart from the use of stem cells, even from burned and debrided skin^205^, future perspectives in the field of TESSs for wound healing are focused on the development of more similar models of artificial skin where 3D bioprinting^211^, designing stem cell niches^212^ or incorporation of immune cells^10,179,180^ will play an important role.

## Conclusion

To date, the positive clinical results obtained with autologous and allogeneic TESSs based on human adult skin cells and hMSCs, regarding successful engraftment (60–90% in most of the studies), safety (slight adverse events in some cases), re-epithelialization and wound healing rates, are promising. However, if we improve current techniques such as the selection of an appropriate animal model and biomaterial or application of 3D bioprinting and expand the toolset with innovative strategies based on ever expanding understanding of skin healing and regeneration (immune cells or stem cell niches), the fabrication of a more functional and physiological TESS, which is clinically beneficial and esthetically acceptable to our patients, is not beyond reach.

## Data Availability

No datasets were generated or analyzed during the current study.
